# The Isoflavone-Rich Fraction of the Crude Extract of the *Puerariae* Flower Increases Oxygen Consumption and BAT UCP1 Expression in High-Fat Diet-Fed Mice

**DOI:** 10.5539/gjhs.v4n5p147

**Published:** 2012-08-12

**Authors:** Tomoyasu Kamiya, Rika Nagamine, Mayu Sameshima-Kamiya, Masahito Tsubata, Motoya Ikeguchi, Kinya Takagaki

**Affiliations:** 1 Research and Development Division, Toyo Shinyaku Co. Ltd., Japan

**Keywords:** *Puerariae* flower extract, kudzu, uncoupling protein 1, brown adipose tissue, isoflavone

## Abstract

*Puerariae* flower extract (PFE) is a crude extract of the Kudzu flower. Previous studies have shown that PFE supplementation exerts anti-obesity and anti-fatty liver effects in high-fat diet-fed mice. In this study, we aimed to identify the PFE components responsible for these effects and to determine their influence on energy expenditure and uncoupling protein 1 (UCP1) expression. Experiments were conducted on C57BL/6J male mice classified into 3 groups: (1) high-fat diet-fed (HFD), (2) high-fat diet-fed given PFE (HFD + PFE), and (3) high-fat diet-fed given the PFE isoflavone-rich fraction (HFD + ISOF). All groups were fed for 42 days. The HFD + PFE and HFD + ISOF groups showed significant resistance to increases in body weight, hepatic triglyceride level, and visceral fat compared to the HFD group. These groups also exhibited significant increases in oxygen consumption and UCP1-positive brown adipose tissue (BAT) area. Our results demonstrate that the active ingredients in PFE are present in the ISOF and that these compounds may increase energy expenditure by upregulation of BAT UCP1 expression. These findings provide valuable information regarding the anti-obesity effects of isoflavones.

## 1. Introduction

*Puerariae* flower extract (PFE) is a crude extract of the Kudzu flower (*Puerariae thomsonii*). It consists of approximately 20% isoflavones as the primary component. Kudzu is a leguminous plant found in Japan, China, and other areas, and its flower is a traditional Chinese medicine frequently used for counteracting symptoms associated with alcohol use, liver injury, and menopause. We previously reported preliminary findings demonstrating the anti-obesity effect of PFE in obese humans (Kamiya et al., 2011). Another clinical study revealed that it reduces visceral fat area with no sexual dimorphism (Kamiya et al., 2012). We also reported that PFE exerts anti-obesity and anti-fatty liver effects in high-fat diet-fed mice. This occurs by upregulating the hormone-sensitive lipase in white adipose tissue (WAT) and the uncoupling protein 1 (UCP1) in brown adipose tissue (BAT) and through suppression of hepatic acetyl-CoA carboxylase (ACC) at the mRNA expression level (Kamiya et al., 2012).

The flower of *Puerariae thomsonii* contains 7 isoflavones: 4 glucosides (tectoridin, tectorigenin 7-O-xylosylglucoside, 6-hydroxygenistein-6,7-diglucoside, and glycitin) and 3 aglycones (tectorigenin, glycitein, and genistein) (Nohara, 2004). Soy isoflavone is reported to exert anti-obesity effects by suppressing lipogenesis in the liver through the increase in protein kinase A activity (Kim et al., 2011) and by promoting lipolysis in WAT through the increase in cAMP levels (Peluso, 2006; Szkudelska et al., 2008). Similarly, it is known that cAMP promotes UCP1 expression in BAT (Rim et al., 2002). Therefore, we hypothesize that the active ingredient of PFE is an isoflavone.

BAT is responsible for non-shivering thermogenesis and diet-induced thermogenesis, which both regulate body temperature and weight (Cannon et al., 1985; Rothwell et al., 1979). Uncoupling protein 1 plays an important role in BAT thermogenesis, and mouse studies have shown a link between UCP1 and obesity (Inokuma et al., 2006). Studies using 18F-fluorodeoxyglucose positron-emission tomographic and computed tomographic scans have revealed that human adults have active BAT (Saito et al., 2009; van Marken Lichtenbelt et al., 2009; Virtanen et al., 2009; Cypess et al., 2009). As previously described, PFE supplementation significantly upregulates UCP1 mRNA expression in BAT; therefore, PFE is expected to also increase energy expenditure.

Here, we conducted an animal study to investigate the effects of the isoflavone-rich fraction (ISOF) of PFE on adipose tissue weight and hepatic triglyceride levels. We also examined the effects of PFE and ISOF on oxygen consumption and UCP1 protein expression levels in BAT.

## 2. Method

### 2.1 Experimental Materials

*Puerariae* flower extract was purchased from Ohta’s Isan Co. Ltd. (Ushiku city, Japan). This compound contains 7 isoflavones: tectoridin (4.70%), tectorigenin 7-*O*-xylosylglucoside (8.37%), 6-hydroxygenistein-6,7-diglucoside (3.38%), glycitin (0.17%), tectorigenin (0.83%), glycitein (0.10%), and genistein (0.06%). All isoflavone standard preparations we used were purchased from either Nagara Science Co., Ltd. (Gifu, Japan) or Tokiwa Phytochemical Co., Ltd. (Chiba, Japan).

### 2.2 Fractionation

**Figure 1:**
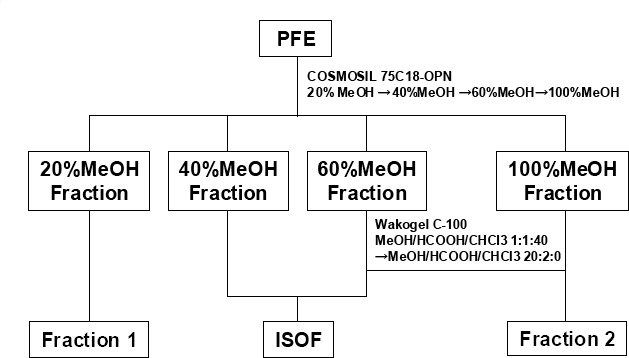
Fractionation flow of ISOF prepared from PFE

The extract was dissolved in 20% MeOH and then sequentially eluted with 20%, 40%, 60%, and finally 100% MeOH for column chromatography (Cosmosil 75C18-OPN, Nacalai Tesque Inc., Kyoto, Japan). The fraction obtained from 20% MeOH was considered to be fraction 1. Next, silica gel column chromatography (solvent A, 1:1:40 [v/v) MeOH:HCOOH:CHCl_3_; solvent B, 10:1 [v/v) MeOH:HCOOH) was performed using the 60% MeOH fraction. The solvent-A-eluted fraction and the 40% MeOH fraction were then mixed to obtain the ISOF. Fraction 2 was obtained by mixing the solvent-B-eluted fraction with the 100% MeOH fraction (Fig. 1). The fractionation yields, calculated as the dry weight of the fraction, were 27.1% for ISOF, 67.4% for fraction 1, and 3.9% for fraction 2.

### 2.3 Quantitative Isoflavone Estimation

The dietary soy isoflavone-aglycone testing method (See Food Safety Notification No. 0823001, August 23, 2006 for guidelines regarding handling of specified health food including soy isoflavones) was modified and implemented to quantify the amount of isoflavones present in PFE and each of the previously described fractions. Each fraction and PFE were dissolved in 50% EtOH and analyzed with high-performance liquid chromatography using a 4.6 × 250 mm YMC-pack ODS-AM-303 column, with UV detection at 264 nm. The flow rate was 1.0 mL/min. Solvent A was CH_3_CN:H_2_O:CH_3_COOH at a ratio of 15:85:0.1 (v/v), and solvent B was CH_3_CN:H_2_O:CH_3_COOH at a ratio of 35:65:0.1. Quantitative isoflavone results are shown in Table 1.

**Table 1:** Results of quantitative isoflavone estimation in each fraction

				(g/100g)
Constituent name	PFE	ISOF	Fraction1	Fraction2
6-Hydroxygenistein 6,7-di-O-glucoside	3.38	12.14	N.D.	N.D.
Glycitin	0.17	0.66	N.D.	N.D.
Tectorigenin 7-O-xylosylglucoside	8.37	30.35	N.D.	N.D.
Genistin	0.27	0.95	N.D.	N.D.
Tectoridin	4.70	16.30	N.D.	1.19
Glycitein	0.10	0.27	N.D.	N.D.
Genistein	0.06	0.16	N.D.	N.D.
Tectorigenin	0.83	2.30	N.D.	N.D.

Total	17.87	63.11	N.D.	1.19

### 2.4 Experimental Animals and Diet

All animal procedures were performed in accordance with the Guidelines for the Care and Use of Experimental Animals of the Japanese Association for Laboratory Animal Science and were approved by the Ethical Committee of Toyo Shinyaku Co., Ltd. Male C57BL/6J mice were purchased from Charles River Laboratories Japan Inc. (Yokohama, Japan) at the age of 6 weeks. At 7 weeks, the mice were divided into 3 groups: (1) high-fat diet (HFD), (2) high-fat diet and given 5% PFE (HFD + PFE), and (3) high-fat diet and given PFE ISOF (HFD + ISOF). All groups were fed for 42 days. For the HFD + ISOF group, 1.355% ISOF from the fractionation yield was used. The animals were kept in an air-conditioned environment with a 12-h light cycle (lights on from 0800–2000). Mice were fed ad libitum during preparatory breeding and were on a controlled feeding regimen during the testing period. During the study, the animals were weighed every 4 days. Food intake was determined every day by subtracting the food remaining in the feed container from the total amount given the day before. The feed composition is shown in Table 2.

**Table 2:** Feed composition

			(g/100g)
	HFD	HFD+PFE	HFD+ISOF
Casein	20.0	20.0	20.0
α-potato starch	28.2	23.2	26.845
Sucrose	13.0	13.0	13.0
Corn oil	20.0	20.0	20.0
Rard	10.0	10.0	10.0
Cellulose	4.0	4.0	4.0
Mineral Mix (AIN-76)	3.5	3.5	3.5
Vitamin Mix (AIN-76)	1.0	1.0	1.0
DL-Methionine	0.3	0.3	0.3
PFE	0.0	5.0	0.0
ISOF	0.0	0.0	1.355
Total	100.0	100.0	100.0

### 2.5 Analysis of Fecal Lipids

Beginning on day 38 (after starting the diet regime), feces were collected for at least 3 days, from which fecal lipids were measured. The feces were dried for at least 3 days at 100°C and their weight was then measured. They were then pulverized and submitted for gross fecal lipid measurement. To determine gross fecal lipid weight, lipid was extracted using the method described by Folch et al. (Folch et al., 1957).

### 2.6 Measurement of Oxygen Consumption and Respiratory Quotient

We measured oxygen consumption and respiratory quotient (RQ) by using an expired-gas analyzer (Oxymax, Columbus Instruments, OH, USA) at days 35–39.

### 2.7 Measurement of Tissue Weight

Forty-two days after the start of the experiment, the mice were sacrificed. The liver, interscapular brown adipose tissue, epididymal, mesenteric, and retroperitoneal adipose tissues were removed and their respective weights measured.

### 2.8 Measurement of Triglycerides in the Liver

The liver was extracted using the method described by Folch et al. (Folch et al., 1957) and was submitted for quantitative triglyceride analysis using an acetylacetone colorimetric method (Fletcher, 1968).

### 2.9 BAT Immunostaining and UCP1-Positive Area Ratio Measurement

The excised BAT was fixed in 10% formalin and sliced after paraffin embedding. The tissues were submitted for UCP1 immunostaining after deparaffinization. They were then processed with a polyclonal anti-UCP1 rabbit antibody (Abcam, Cambridge, UK) according to the avidin-biotin-peroxidase complex method. Based on the method of Cinti et al. (Cinti et al., 2002), the UCP1-positive area was calculated as follows: digital images were captured using a camera installed in the optical microscope (IX-70, Olympus Corporation, Tokyo, Japan), and the UCP1-positive area was measured using image analysis software (WinROOF V5.6, Mitani Corporation, Tokyo, Japan). The UCP1-positive area in the digital image was divided by the total tissue area to calculate the UCP1-positive area ratio.

### 2.10 Statistical Analysis

Data were expressed as the mean ± SEM. For comparisons between groups, Fisher’s protected least significance difference (PLSD) test was used. All statistical analyses were performed using Statview (version 5.0, SAS Institute Japan Ltd., Tokyo, Japan), and significance was set at *p* < 0.05.

## 3. Results

### 3.1 Food Intake, Body Weight, Adipose Tissue Weight, and Fecal Lipid Levels

Final body weight, weight gain, and white and brown adipose tissue weights were significantly lower in the HFD + PFE group compared to the HFD group (Table 3). This suggests that PFE inhibits the fat weight gain caused by a high-fat diet. The HFD + ISOF group had similar results as the HFD + PFE group, and were not significantly different. This suggests that the active ingredient in PFE preventing weigh gain is also present in the ISOF. There were no significant differences in food intake or fecal lipid levels between the HFD + PFE and HFD + ISOF groups compared to the HFD. Because of this, the anti-obesity effects of PFE and ISOF are likely not due to differences in energy intake.

**Table 3:** Food intake, body weight, adipose tissue relative weights, fecal lipid, and hepatic triglyceride in male C57BL/6J mice fed HFD, HFD+PFE, and HFD+ISOF diets for 42 day

	HFD	HFD+PFE	HFD+ISOF
Food intake, g/day	2.8±0.1	2.6±0.1	2.7±0.0
Final body weight, g	31.5±0.7 a	27.3±0.6 b	28.2±0.4 b
Body weight gain, g	7.3±0.8 a	3.3±0.3 b	3.9±0.4 b
White adipose tissue weight			
Epididymal, g/100g body weight	5.2±0.3 a	2.7±0.2 b	3.3±0.2 b
Mesentric, g/100g body weight	1.4±0.1 a	0.8±0.1 b	0.9±0.0 b
Retroperitoneal, g/100g body weight	0.4±0.0 a	0.2±0.0 b	0.2±0.0 b
Total, g/100g body weight	6.9±0.4 a	3.8±0.3 b	4.5±0.2 b
Brown adipose tissue weight, g/100g body weight	1.0±0.0 a	0.6±0.0 b	0.7±0.0 b
Fecal lipid, g/day	0.022±0.002	0.025±0.003	0.023±0.004
Hepatic triglyceride, mg/g wet tissue	46.7±3.7 a	31.5±2.5 b	26.2±1.5 b

Note: The data represent the mean ± SEM values (n = 7-8). Different symbols represent *p*<0.05 as compared with the other groups

### 3.2 Hepatic Triglyceride Levels

Hepatic triglyceride levels in both the HFD + PFE and HFD + ISOF groups were significantly lower than those in the HFD group (Table 3).

### 3.3 Oxygen Consumption and Respiratory Quotient

**Figure 2:**
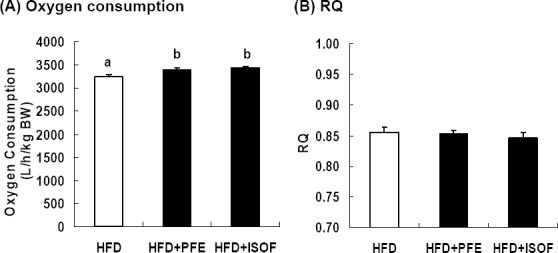
Effects of PFE and ISOF on oxygen consumption and RQ The data represent the mean ± SEM values (n = 7-8). Different symbols represent *p*<0.05 as compared with the other groups

Figure 2 shows oxygen consumption and RQ on days 35–39 after the start of the experiment. As compared to the HFD group, the HFD + PFE and HFD + ISOF groups had significantly higher oxygen consumption but no significant differences in RQ. These results suggest that PFE and ISOF increase energy consumption without affecting RQ.

### 3.4 BAT Immunostaining and UCP1-Positive Area Ratio

**Figure 3:**
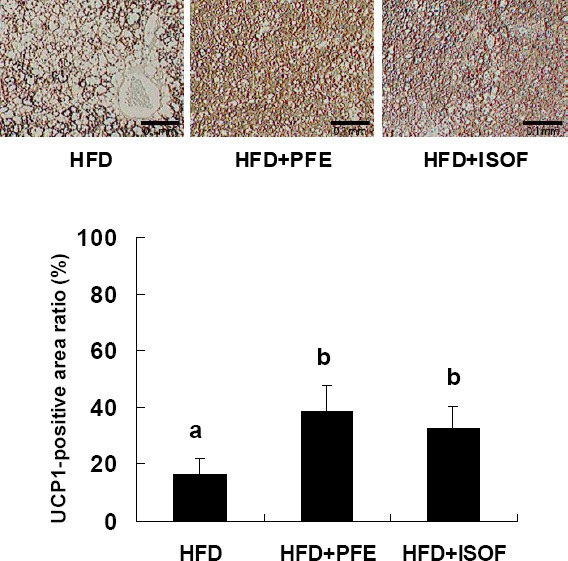
UCP1 immunohistochemical staining and UCP1-positive area ratio in BAT The data represent the mean ± SEM values (n = 6-8). Different symbols represent *p*<0.05 as compared with the other groups

The HFD + PFE and HFD + ISOF groups had significantly higher UCP1-positive area ratios than the HFD group (Fig. 3). The HFD + PFE and HFD + ISOF groups also showed an apparent inhibition to fat accumulation in BAT. The BAT weight was significantly lower in both the HFD + PFE and HFD + ISOF groups compared to the HFD group (Table 3), likely due to reduced fat accumulation.

## 4. Discussion

It has been reported that soy isoflavones, such as genistein and daidzein, exert anti-obesity effects (Kim et al., 2005; Lephart et al., 2004). Recently, Kim et al. demonstrated that daidzein supplementation prevented obesity and non-alcoholic fatty liver disease in an animal study (Kim et al., 2011). It is therefore believed that isoflavones may have the potential to prevent obesity and fatty liver disease. It has also been reported that genistein down-regulates ACC mRNA expression in HepG2 cells and stimulates glycerol release in isolated rat adipocytes (Shin et al., 2007; Szkudelska et al., 2000). In an earlier study, we showed that PFE exerts similar effects as these soy isoflavones in high-fat diet-fed mice (Kamiya et al., 2012).

The present study indicates that dietary PFE and its isoflavone-rich fraction both produce anti-obesity effects in high-fat diet-fed mice. There were no significant differences between the HFD + PFE and HFD + ISOF groups in final body weight, weight gain, or white adipose tissue weight. Similarly, for these groups there were no significant differences in the amount of isoflavone intake (23.4 ± 1.0 mg/day for HFD + PFE and 23.4 ± 0.3 mg/day for HFD + ISOF), as calculated from the isoflavone content of PFE and ISOF (Table 1), dietary composition (Table 2), and amount of food intake (Table 3). Accordingly, our findings suggest that the active ingredients responsible for the PFE anti-obesity effect are indeed isoflavones.

Lephart et al. (Lephart et al., 2004) reported that supplementation with a soy isoflavone mixture significantly upregulated UCP1 mRNA expression in the BAT of Long-Evans rats. Additionally, we reported that PFE supplementation significantly upregulated UCP1 mRNA expression in BAT (Kamiya et al., 2012). Uncoupling protein 1 is a key factor that determines the level of thermogenesis in BAT, and a number of mice studies have revealed that it controls body fat levels by promoting energy expenditure (Inokuma et al., 2006; Ricquier et al., 2000; Saito et al., 1985). Brown adipose tissue promotes the hydrolysis of stored triglycerides by endogenous lipases, leading to the mobilization of fatty acid as fuel for thermogenesis. Cyclic-AMP promotes lipolysis and UCP1 expression, which are important factors for BAT thermogenesis, and activates cAMP-dependent protein kinase (PKA), which promotes lipolysis of triglycerides. Moreover, the cAMP response element binding protein (CREBP) increases UCP1 expression (Rim et al., 2002). Flavonoids, such as genistein and quercetin, increase cAMP in adipocytes (Peluso, 2006; Kuppusamy et al., 1994), but there have been no in vivo studies on the effects of soy isoflavones on oxygen consumption. In the current study, PFE and ISOF significantly increased UCP1 expression in BAT as well as oxygen consumption. Supplementation with PFE did not significantly upregulate gene expression related to beta oxidation (Kamiya et al., 2012); therefore, ISOF-induced increases in energy expenditure may be largely due to upregulation of BAT UCP1 expression. These findings provide valuable information regarding the anti-obesity effects of isoflavones.

The oxygen consumption of the HFD + PFE and HFD + ISOF groups were significantly higher than the HFD group, 4.7% and 6.0% respectively (Fig 1). C57BL/6J mice reportedly use 6–7 kcal per day (Li et al., 2010); therefore, PFE and ISOF supplementation seem to increase energy expenditure ~15 kcal over 42 days. Since adipose tissue contains 7 kcal/g, PFE and ISOF supplementation led to ~2 g of WAT reduction in these mice. In this study, the WAT weights of the HFD + PFE and HFD + ISOF groups (-2.4 g and -3.1 g, respectively) were significantly lower than that of the HFD group (Table 3). These results suggest that PFE and ISOF-induced increases in energy expenditure produce significant WAT reductions.

Within PFE there are 4 major types of isoflavones: 6-hydroxygenistein 6,7-di-O-glucoside, tectorigenin 7-O-xylosylglucoside, tectoridin, and tectorigenin (Table 1). When cultured with enteric bacteria, tectoridin and tectorigenin-7-O-xylosylglucoside are metabolized into tectorigenin, and 6-hydroxygenistein 6,7-di-O-glucoside is metabolized into 6-hydroxygenistein (an aglycone isoflavone) (Tsuchihashi et al., 2009; Hirayama et al., 2011). Tectorigenin is detected in the urine after oral administration of PFE in human subjects (unpublished data). Consequently, the major isoflavones of PFE are likely absorbed into the bloodstream in tectorigenin form. As previously described, we have reported that PFE supplementation significantly suppresses hepatic ACC, the rate-limiting enzyme in fatty-acid biosynthesis (Kamiya et al., 2012). Moreover, our recent unpublished data show that tectorigenin suppresses hepatic lipid accumulation in HepG2 cells. In the present study, hepatic triglyceride levels of both the HFD + PFE and HFD + ISOF groups were significantly lower than that in the HFD group (Table 3). This result may be caused by suppression of hepatic lipogenesis; however, future studies are required to determine the underlying mechanisms responsible for this.

Hepatic lipogenesis is an important factor in promoting triglyceride accumulation in WAT and the overall anti-obesity effect of PFE. It has been reported that increased hepatic lipogenesis contributes to fat accumulation in WAT by increasing VLDL secretion from the liver (Yamazaki et al., 2005). In another study, subjects with established obesity showed increased hepatic lipogenesis that may have contributed to their excessive fat mass (Diraison et al., 2002). Accordingly, the anti-obesity actions of PFE seem to be due to both the upregulation of energy expenditure as well as the suppression of lipogenesis in the liver.

In summary, we conducted an animal study to investigate the effect of PFE and ISOF on adipose tissue weight, energy expenditure, and BAT UCP1 expression. As a result, the active ingredient causing the anti-obesity effect of PFE is thought to be an isoflavone. In addition, PFE and ISOF also increased energy expenditure, which is mainly attributed to the upregulation of BAT UCP1 expression. In future studies, we will isolate the single active ingredient of this anti-obesity effect of PFE.
